# In Vivo Autophagy Up-Regulation of Small Intestine Enterocytes in Chinese Soft-Shelled Turtles during Hibernation

**DOI:** 10.3390/biom9110682

**Published:** 2019-11-01

**Authors:** Waseem Ali Vistro, Yue Zhang, Xuebing Bai, Ping Yang, Yufei Huang, Wenjia Qu, Abdul Sattar Baloch, Ruizhi Wu, Imran Tarique, Qiusheng Chen

**Affiliations:** MOE Joint International Research Laboratory of Animal Health and Food safety, College of Veterinary Medicine, Nanjing Agricultural University, Nanjing 210095, China; 2017207039@njau.edu.cn (W.A.V.); 2018807113@njau.edu.cn (Y.Z.); 2016107003@njau.edu.cn (X.B.); yangping@njau.edu.cn (P.Y.); 2017207007@njau.edu.cn (Y.H.); 2018807169@njau.edu.cn (W.Q.); balochsattar@ymail.com (A.S.B.); 2016107004@njau.edu.cn (R.W.); samoo_imran88@hotmail.com (I.T.)

**Keywords:** enterocytes, autophagy, autophagosome, ATG7, LC3, p62, hibernation, Chinese soft-shelled turtle

## Abstract

Many studies have focused on how autophagy plays an important role in intestinal homeostasis under pathological conditions. However, its role in the intestine during hibernation remains unclear. In the current study, we characterized in vivo up-regulation of autophagy in enterocytes of the small intestine of Chinese soft-shelled turtles during hibernation. Autophagy-specific markers were used to confirm the existence of autophagy in enterocytes through immunohistochemistry (IHC), immunofluorescence (IF), and immunoblotting. IHC staining indicated strong, positive immunoreactivity of the autophagy-related gene (ATG7), microtubule-associated protein light chain (LC3), and lysosomal-associated membrane protein 1 (LAMP1) within the mucosal surface during hibernation and poor expression during nonhibernation. IF staining results showed the opposite tendency for ATG7, LC3, and sequestosome 1 (p62). During hibernation ATG7 and LC3 showed strong, positive immunosignaling within the mucosal surface, while p62 showed strong, positive immunosignaling during nonhibernation. Similar findings were confirmed by immunoblotting. Moreover, the ultrastructural components of autophagy in enterocytes were revealed by transmission electron microscopy (TEM). During hibernation, the cumulative formation of phagophores and autophagosomes were closely associated with well-developed rough endoplasmic reticulum in enterocytes. These autophagosomes overlapped with lysosomes, multivesicular bodies, and degraded mitochondria to facilitate the formation of autophagolysosome, amphisomes, and mitophagy in enterocytes. Immunoblotting showed the expression level of PTEN-induced kinase 1 (PINK1), and adenosine monophosphate-activated protein kinase (AMPK) was enhanced during hibernation. Furthermore, the exosome secretion pathway of early–late endosomes and multivesicular bodies were closely linked with autophagosomes in enterocytes during hibernation. These findings suggest that the entrance into hibernation is a main challenge for reptiles to maintain homeostasis and cellular quality control in the intestine.

## 1. Introduction

Energy-saving strategies are used by different kinds of animals to survive in stressful and food-scarce environmental conditions. One of the strategies, hibernation, is a remarkable physiological circumstance described by a profound yet reversible rest-like state.

Hibernation is an adaptive survival strategy in response to cold and foodless winter seasons, mostly found in mammals and birds [1]. Previously, our research group addressed the life cycle of the Chinese soft-shelled turtle that consists of a hibernation period, which starts from the early days of December and continues into March, and a nonhibernation period from May to October [2,3]. Hibernation is a way that some animals, including Chinese soft-shelled turtles, cope with unfavorable ecological conditions by dramatically reducing their body activity [4,5], metabolic rate [6], and physiological parameters such as body temperature and heart and respiratory rate compared to nonhibernation [7,8]. Hibernation is an energy-limiting period [6], as during this period the animals are sedentary, and the demand for oxygen is reduced severely, thus eliminating the need for food during cold seasons [2,9]; although, animals face different kinds of challenges in these critical situations. However, animal bodies have developed different physiological defense mechanisms against any stressor that could enter into the animals along with nutrients in the digestive system [10]. One of the defense mechanisms is autophagy, which participates in the promotion of cellular fitness and the maintenance of intestinal homeostasis [11].

Autophagy is a degradation pathway that is stimulated by cellular or environmental stresses in order to remove damaged organelles, protein aggregates, and intracellular pathogens through the formation of double-membrane structures, called autophagosomes [12], which, when fused with lysosomes, deliver their contents for degradation by lysosomal enzymes [13]. This process comprises the intensive action of numerous cytoplasmic proteins [14]. Autophagy performs essential roles in the maintenance of cellular and tissue homeostasis by degrading cell contents under the condition of nutrient starvation [15]. Besides that, autophagy plays an important role in numerous physiological and pathological conditions such as aging, immunity, cancer, inflammatory diseases, metabolic stress [12,16,17,18], and intestinal cell survival during physiological stress [19]. Among intestinal diseases, autophagy was first linked to pathogenesis of Crohn’s disease when genome-wide association studies identified mutations in the autophagy-related gene *ATG16L1* as a risk factor for Crohn’s disease [20]. Recently, researchers have observed the role of autophagy in dendrite epithelial cell communication, adaptive immunity response, NOD2-directed bacteria sensing, lysosome destruction, and immune-mediated clearance to be important for inflammatory bowel disease (IBD) pathogenesis [21,22]. Autophagy has been shown to be critical for the recognition and degradation of pathogens, thus functioning as an innate barrier against infection [23]; bacteria targets include *Listeria monocytogenes* [24] and *Salmonella typhimurium* [25]. Despite the many studies in which autophagy responds to provide defense mechanisms against pathological conditions, the autophagic pathway in the small intestine of reptiles during hibernation remains obscure.

The known biology of autophagy was revolutionized following the identification of autophagy genes within mammals [26]. ATG7 is the most essential member of an autophagy-related gene family that encodes the E1-like enzyme, which facilitates both LC3 and other autophagy-related genes [27]. The microtubule-associated protein light chain (LC3) and p62 biomarkers are regularly used for checking the intensity of autophagy [28]. LC3 is mostly used for to check the autophagic activity in the milieu of cellular housekeeping and autophagic cell death [29]. LC3 is a soluble protein that is recruited from the cytoplasm to the autophagosome membrane. During autophagy, LC3-I is conjugated to phosphtidylethanolamine to form LC3-phosphatidylethanolamine conjugate (LC3-II), which is strongly bound to the autophagosomal membrane [30]. The p62 proteins, also called sequestosome1 (SQSTM-1), help with acknowledgment of autophagic cargo [31]. LAMP1 is a well-known protein for lysosomal/autophagosome markers [32]. PTEN-induced kinase 1 (PINK1) is the specific marker of mitophagy [33]. Adenosine monophosphate-activated protein kinase (AMPK) is one of the most important molecular energy sensors in eukaryotic cells [34]. AMPK is required for the induction and progression of the autophagy process. AMPK regulates many metabolic processes. One of the catabolic processes activated by AMPK is macroautophagy (here after, autophagy) [35].

Autophagy is a conserved pathway among vertebrates and is well-studied in mammals [36]. It also contributes in starvation and various extrinsic and intrinsic stresses. However, its role in reptilian enterocytes has not yet been reported. Chinese soft-shelled turtles are one of the most nutritionally and pharmacologically important animals in China. As a seasonal hibernating animal, this species is an excellent model for studying the regulation of this natural protective mechanism. Therefore, we have made a hypothesis concerning in vivo up-regulation of autophagy in enterocytes of the small intestine of the Chinese soft-shelled turtle during hibernation.

## 2. Materials and Methods

### 2.1. Animals and Tissue Preparation

All techniques with the Chinese soft-shelled turtles (*Pelodiscus sinensis*) were directed according to the Animal Research Institute Committee guidelines of Nanjing Agricultural University, China. A total of 20 mature *P. sinensis,* aged between of 4 and 5 years, were selected for the current research. The turtles were brought from an aquatic pond in Nanjing, Jiangsu Province of China during the hibernation period in the months of February and March (n = 10) and the nonhibernation period in the months of August and September (n = 10). The average body weight (mean ± SD) of each mature *P. sinensis* was 1.45 ± 0.10 kg. Before sampling, the temperature was noted during hibernation (4 to 8 °C) and nonhibernation periods (20 to 25 °C). We fed the turtles in the nonhibernation period and did not feed the turtles in the hibernation period. After at least 24 h, the animals were anaesthetized using intraperitoneal injection of pentobarbital sodium (20 mg kg^−1^) and were euthanized by neck dislocation. The middle part (jejunum) of the small intestine was collected and preserved quickly for experiments (details below). All the protocols were approved by the Science and Technology Agency of Jiangsu Province (SYXK (SU) 2017-0007).

### 2.2. Immunohistochemistry

Small intestinal tissue slides of both groups (hibernation and nonhibernation) were deparaffinized in xylene for 10 min two times. All slides were exposed in a grading series of ethanol (75–100%) and kept for 2 min in each grade. Antigenic sites were exposed by boiling for 2–3 min in 30% sodium citrate and then rinsed three times in phosphate-buffered saline (PBS) for 5 min. These sections were covered with 3% hydrogen peroxide in PBS for 15 min at 37 °C in order to block the further activity of endogenous peroxidase. The samples were blocked with 5% bovine serum albumin and incubated with primary antibody (Table 1) in a moisture chamber at 4 °C overnight. After washing, the sections were incubated with secondary antibody biotinylated anti-rabbit IgG (Boster Bio-Technology, Wuhan, China) for 60 min at 37 °C. Then, they were rehydrated in PBS (pH 7.2) and incubated with avidin-biotinylated peroxidase complex for 45 min at 37 °C. After washing with PBS, peroxidase activity was exposed using DAB (Boster-BIO-technology, Wuhan, China) according to manufacturer guidelines.

### 2.3. Immunofluorescence

Tissue paraffin sections of the small intestine (hibernation and nonhibernation) were incubated with primary antibody at 4 °C for 24 h (Table 1). Then, secondary antibody was applied for 60 min at 37 °C. Samples were fixed with mounting medium containing 4’,6-diamidino-2-phenylindole (DAPI) to stain the nucleus. Immediately thereafter, images were captured with a BX53 camera (DP73, Olympus, Tokyo, Japan).

### 2.4. Immunoblotting

Small intestinal tissue samples were homogenized in ice-cold radio immunoprecipitation assay (RIPA) buffer that contained protease and phosphate inhibitors (Roche Applied Science, Penzberg, Germany). After centrifugation at 13,000× *g* for 15 min, the protein concentration in the supernatant was quantified with the bicinchoninic acid protein assay (Thermo Scientified). Protein samples (45 µg) were loaded on 4–12% SDS–PAGE gels. Electrophoresis was run at 120 V for 2 h at 4 °C (Bio-Red Mini-Protean), and immunoblotting was performed with primary antibodies (diluted 1:1000) and β-actin (1:10,000; Bioworld, Nanjing, China). The intensities of target proteins LC3, p62, PINK1, and AMPK were normalized against β-actin. Three independent experiments were performed for western blotting quantification analysis.

### 2.5. Transmission Electron Microscopy

After tissue sample collection, samples were cut in to small pieces, immersed in 2.5% glutaraldehyde solution, and then fixed in 1% hungry acid for transmission electron microscopy experiments. Samples were dehydrated with gradient alcohol, which was replaced with acetone, and embedded in Epon812, double stained with uranyl acetate citrate, and observed by HITA H-7650 transmission electron microscopy (TEM).

### 2.6. Statistical Analysis

ImageJ software was used for the measurement [37] of immunoblotting protein bands and immunofluorescence intensity of villi in the small intestine, and data were reported as the mean ± SEM. A *t*-test was performed using GraphPad Prism to determine significant differences between the two groups. The differences were considered significant at *P* ˂ 0.05 (one-tailed).

## 3. Results

### 3.1. Cellular Localization of Different Autophagy Protein Markers in the Small Intestine During Hibernation

**Figure 1:**
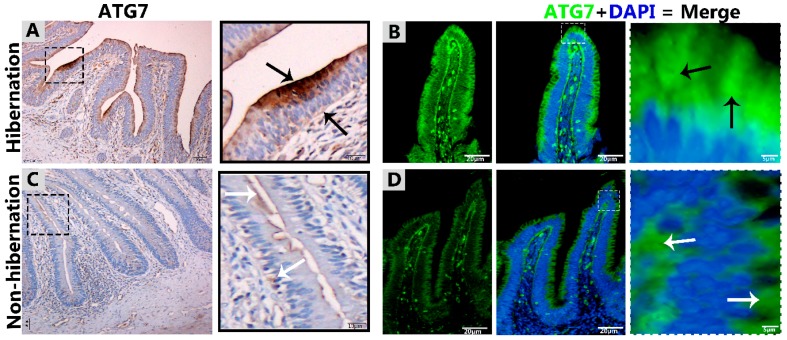
Immunohistochemistry and immunofluorescence staining of ATG7 in the mucosal surface of the small intestine of *Pelodiscus sinensis*. During hibernation (**A**,**B**) and nonhibernation (**C**,**D**), there were strong, positive (black arrow) and weak expressions (white arrow). Scale bar = 50 µm (**A**,**C**) and 20 µm (**B**,**D**).

**Figure 2:**
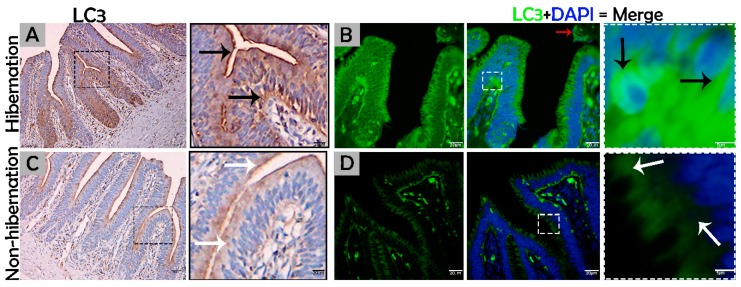
Immunohistochemistry and immunofluorescence staining of LC3 in the mucosal surface of the small intestine of *P. sinensis.* During hibernation (**A**,**B**) and nonhibernation (**C**,**D**), there was a strong, positive expression (black arrow), a strong, positive expression in the lumen of the intestine (red arrow), and weak expression (white expression). Scale bar = 50 µm (**A**,**C**) and 20 µm (**B**,**D**).

**Figure 3:**
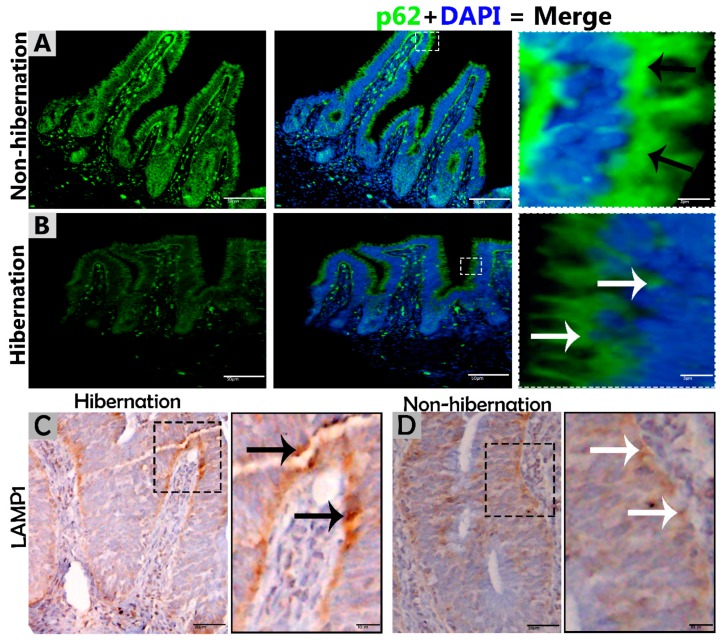
Immunofluorescence staining of p62 and immunohistochemistry of LAMP1 in the mucosal surface of the small intestine of *P. sinensis.* During hibernation (**B**,**C**) and nonhibernation (**A**,**D**), there were strong, positive (black arrow) and weak expressions (white arrow). Scale bar = 50 µm (**A**,**B**) and 20 µm (**C**,**D**).

**Figure 4:**
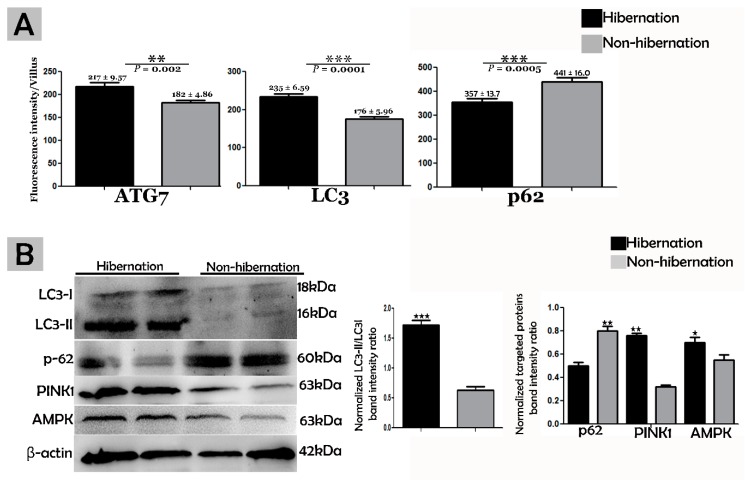
(**A**) Fluorescence intensity quantification of ATG7, LC3, and p62 (n = 10 turtle/group and 20 villi/section of each turtle). The values represent mean ± SEM. * indicates a statically significant difference between hibernation and nonhibernation. (**B**) Immunoblotting protein expression levels of LC3, p62, PINK1, and AMPK in the small intestine of *P. sinensis* during hibernation and nonhibernation. Experiments were repeated three times, with similar results in each.

To investigate the protein marker expressions related to autophagy during hibernation of *P. sinensis,* immunohistochemistry (IHC) analyses were performed to perceive the positive reactions of ATG7, LC3, and LAMP1 in turtle intestines. In contrast to nonhibernation (Figure 1C, Figure 2C and Figure 3D), the enterocytes during hibernation showed strong, positive immunoreactivities of ATG7, LC3, and LAMP1, especially at apical and basal regions of the mucosal surface (Figure 1A, Figure 2A, Figure 3C). Furthermore, immunofluorescence (IF) staining was performed to determine the localization of ATG7, LC3, and p62 proteins. The IF staining results showed the opposite tendency for ATG7, LC3, and p62 proteins. Strong, positive immunosignaling of ATG7 and LC3 was detected at apical and basal regions of the mucosal surface during hibernation (Figure 1B and Figure 2B); however, weak immunosignaling was detected during nonhibernation (Figure 1D and Figure 2D). While during nonhibernation p62 showed strong, positive immunosignaling at apical and basal regions of the mucosal surface, it showed weak immunosignaling during hibernation (Figure 3A,B). Statistical analysis proved that the quantification of immunofluorescence intensity of ATG7 and LC3 was significantly increased during hibernation. However, p62 significantly increased during nonhibernation as compared to hibernation (Figure 4A). Similarly, immunoblotting analysis confirmed the protein levels of LC3 and AMPK were enhanced during hibernation as compared to nonhibernation, and the protein level of p62 increased during nonhibernation rather than during hibernation (Figure 4B). Finally, the autophagy-specific markers, ATG7, LC3, p62, and LAMP1, and AMPK progression of the signaling autophagy proteins analyzed through IHC, IF, and immunoblotting proved that the autophagic pathway was up-regulated in the enterocytes of the small intestine during hibernation. Furthermore, the dispersion patterns of these autophagic proteins in the mucosal region of the small intestine are summarized in Table 2 to elucidate the similarities and dissimilarities during hibernation and nonhibernation.

### 3.2. Ultrastructural Formation and Localization of Autophagosomes in Enterocytes of Small Intestine During Hibernation

**Figure 5:**
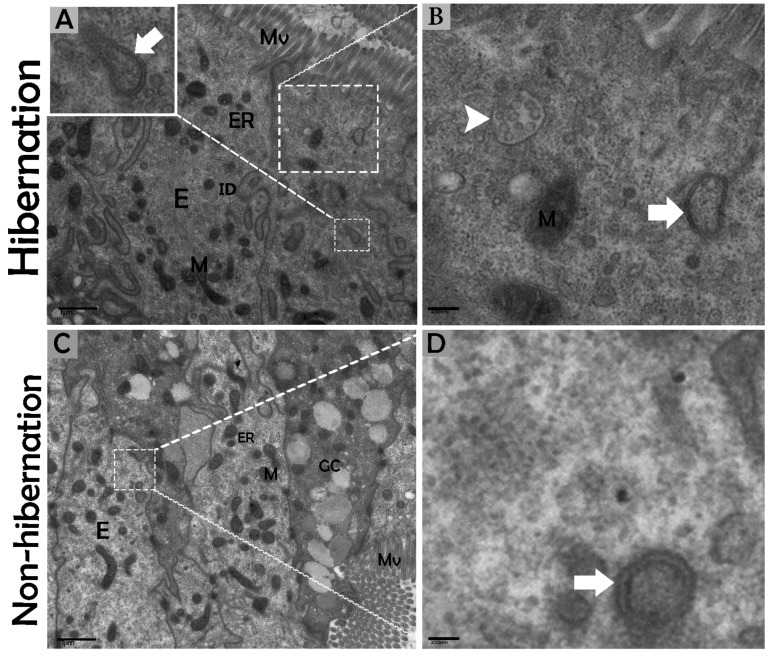
Formation of phagophores in enterocytes of the small intestine in *P. sinensis* during hibernation (**A**,**B**) and nonhibernation periods (**C**,**D**). E, enterocyte; GC, goblet cell; M, mitochondria; ER, endoplasmic reticulum; ID, interdigitation; multivesicular bodies (white arrow head), phagophore (white bold arrow). Scale bar = 1 µm (**A**,**C**), 400 nm (**B**), and 200 nm (**D**).

**Figure 6:**
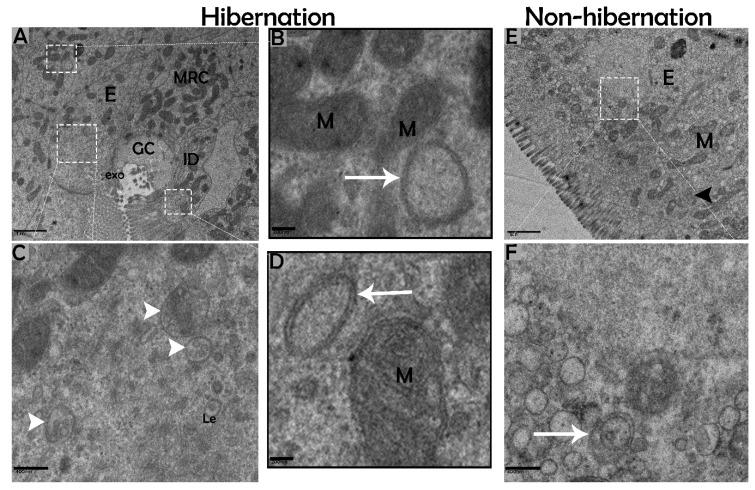
Formation of autophagosomes in enterocytes of the small intestine in *P. sinensis* during hibernation (**A**–**D**) and nonhibernation periods (**E**,**F**). E, enterocyte; GC, goblet cell; MRC, mitochondria-rich cells; M, mitochondria; autophagosome (white arrow); interdigitation (black arrow head); ee, early endosome; Le, late endosome; multivesicular bodies (white arrow head); exo, exosome. Scale bar = 1 µm (**A**,**E**), 200 nm (**B**,**D**), and 400 nm (**C**,**F**).

**Figure 7:**
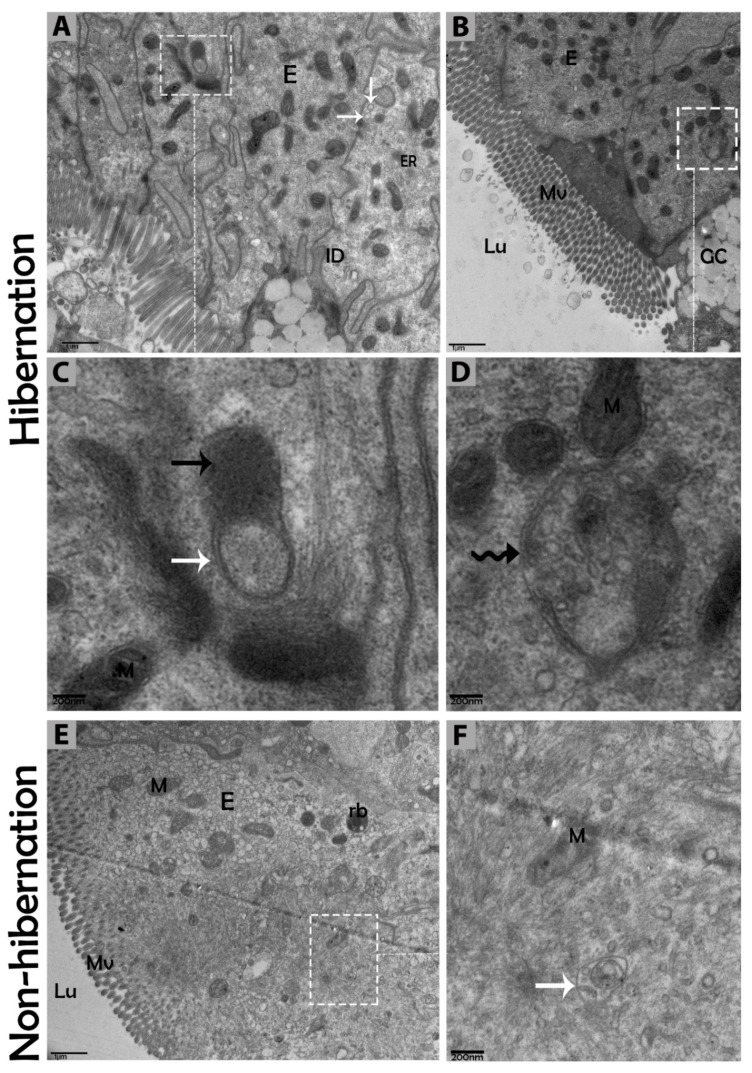
Autophagolysosome and amphisome formation in enterocytes of the small intestine in *P. sinensis* during hibernation (**A**–**D**) and nonhibernation periods (**E**,**F**). E, enterocyte; GC, goblet cell; M, mitochondria; Lu, lumen; Mv, microvilli; rb, residual bodies; autophagosome (white arrow), lysosome (black arrow); amphisome (curve black arrow). Scale bar = 1 µm (**A**,**B**,**E**) and 200 nm (**C**,**D**, **F**).

**Figure 8:**
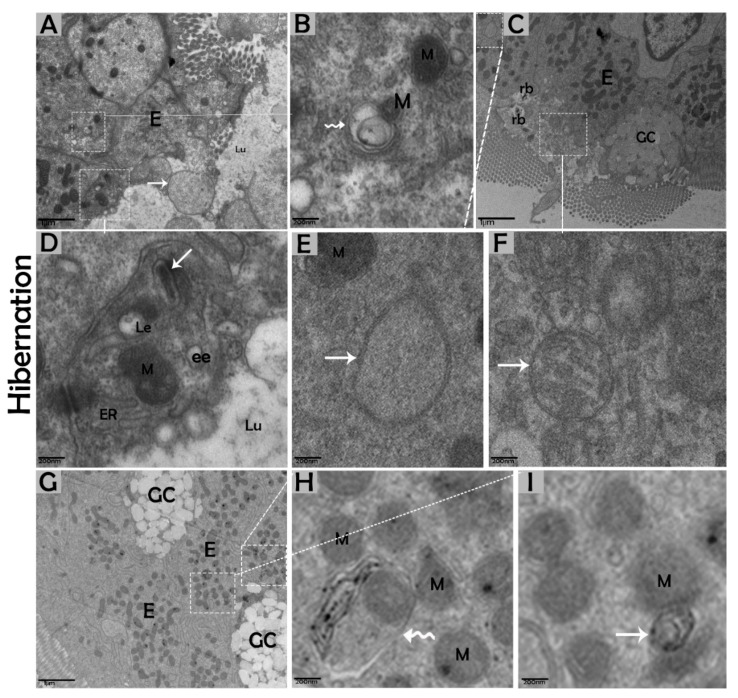
Luminal formation of autophagosomes and mitophagy in enterocytes of the small intestine in *P. sinensis* during hibernation (**A**–**I**). E, enterocyte; GC, goblet cell; M, mitochondria; ER, endoplasmic reticulum; rb, residual bodies; autophagosome (white arrow); mitophagy (curve white arrow); ee, early endosome; Le, late endosome; Lu, lumen. Scale bar = 1 µm (**A**,**C**,**G**) and 200 nm (**B**,**D**,**E**–**I**).

The biological process of autophagy was detected in the cytoplasm of enterocytes of the small intestine in *P. sinensis* during hibernation. TEM findings revealed that during hibernation, the cytoplasm of enterocytes exhibited the cumulative formation of a double-membraned, cup-shaped structure, called a phagophore, and a well-developed endoplasmic reticulum (Figure 5A–D) appeared as the first step of autophagy. After the closure of the blind ends of the phagophore, the autophagosomes with organelles enclosed inside were observed (Figure 6A,B,D,F and Figure 7A). The fusion of the autophagosome with lysosomes caused development of autophagolysosomes (Figure 7C), and in the lysosomal pathways, the multivesicular bodies (MVBs) were engulfed by autophagosomes to form amphisomes (Figure 7D). However, no evidence was found for the formation of autophagolysosomes and amphisomes in enterocytes during nonhibernation (Figure 7E,F). Additionally, mitophagy is the main type of autophagy; during hibernation, many degraded mitochondria were closely associated and overlapped with autophagosomes, suggesting that more mitophagy occurred during hibernation (Figure 6B,D and Figure 8B,D,G,H,I). Furthermore, we detected a mitophagy-related protein, PINK1, which regulates mitophagy by changing the membrane potential of mitochondria. Immunoblotting showed that the expression level of PINK1 was enhanced during hibernation as compared to nonhibernation (Figure 4B). As the final step of autophagy, many residual bodies with electron-dense contents of digested organelles were closely associated with autophagosomes in enterocytes during hibernation (Figure 8C). During hibernation, the apical membranes of enterocytes broke, and the cytoplasm along with the remains of organelles and autophagosomes were discharged into the lumen of the small intestine (Figure 8A,C,F). A similar finding was also observed in IF staining of the LC3 marker of autophagy during hibernation (red arrow) (Figure 2B). These observations proved that the autophagic pathway was significantly dominant inside the cytoplasm of enterocytes as well in the lumen of the small intestine of *P. sinensis* during hibernation. Besides that, during hibernation, the cytoplasm of enterocytes exhibited the exosomes secretion pathway, as early endosomes, late endosomes, and multivesicular bodies were closely associated with autophagosomes (Figure 6C and Figure 8D). Finally, we summarized the subcellular features of autophagy in enterocytes of the small intestine during hibernation and nonhibernation (Table 3).

## 4. Discussion

The gastrointestinal tract response to long-term aphagia has been studied in estivating amphibians [38] and hibernating mammals [39]. Although the time of fasting in endothermic animals is in the range of a few hours, in ectotherms, it can be several months (characteristic of hibernation) [40]. During the hibernation periods of mammals, amphibians, and reptiles, the efficient removal of cytoplasmic contents are thought to be important for cellular life and the maintenance of the internal milieu; however, this mechanism is poorly understood [4,40]. Previous reports have suggested that the removal of residual material and cytoplasm are mediated by intestinal epithelial cells through engulfment [5]. However, hibernating animals protect their internal body physiology through different mechanisms [41]. One of them is autophagy. However, the role of autophagy in the small intestine of ectotherms is largely unknown. In our current study, we investigated the in vivo role of autophagy in enterocytes of the small intestine of *P. sinensis* during hibernation. Our IHC and IF results for ATG7 and LC3 showed strong, positive expressions at luminal and basal regions of the mucosal surface of the small intestine during hibernation. These expression patterns of different proteins of autophagy might be involved in the maintenance of intestinal homeostasis. Nighot et al. [42] reported similar patterns of expression during cell starvation of ATG7, LC3, and p62 in intestinal epithelial cells, which enhanced the tight junction barrier function by targeting the degradation of the CLDN-2 pore-forming protein. Increasing the expression of CLDN-2 caused the epithelial monolayer to become leaky and the conductivity of Na^+^ to increase [43,44]. After the stimulation of autophagy during starvation, the gap junction connexin proteins (CX50 and CX43) were targeted by the LC3 protein in Hela cells [45]. Furthermore, in contrast to a previous report [46], we detected weak expression of p62 in the small intestine during hibernation in our results. A previous report confirmed the enrichment of autophagy with the LC3 level in aggregation with decreased p62 levels [47]. In our results, ATG7, LC3, and p62 showed the opposite tendency to each other, which was an indication of active autophagy [48]. The p62 protein functions as a selective autophagy receptor for the degradation of autophagic cargo and serves as an index of autophagy degradation [37,49]. Consequently, these findings suggested that, during the hibernation period, these autophagic proteins promoted the tight junction barrier function of intestinal epithelial cells, which is essential for maintenance of intestinal homeostasis. Nutrient starvation-induced autophagy significantly increased trans-epithelial electrical resistance and decreased the ratio of Na^+^/K^+^ paracellular permeability [50]. The knockout of ATG7 in intestinal epithelial cells of mice synergistically intensified the intestinal disease, increased widespread submucosal inflammation [51], and inhibited the secretion of intestinal mucins [52]. These findings strongly suggest that ATG7 is essential for controlling the intestinal inflammatory response and defense mechanism against intestinal pathogens. In addition, in our results the immunoreactivity of LAMP1 was increased during hibernation as compared to nonhibernation. LAMP1 is a lysosomal marker. An in vitro study using melanoma cells concluded that LAMP1 is used for regulation of carbohydrates [53]. However, the lysosome is the last point of many vesicle-trafficking pathways, including those for phagocytosis and autophagy [54].

We further characterized the autophagic response of intestinal enterocytes using immunoblotting and TEM. TEM is a reliable and accurate way of monitoring autophagy [55]. In the current study, the expression level of AMPK was increased, and the formation of phagophores and double-membrane structures, called autophagosomes [13], were enhanced in enterocytes of the small intestine during hibernation. AMPK is activated selectively by glucose deprivation [35]. AMPK increases an autophagic flux by contributing to autophagosome maturation and enhances the progression of autophagy [56]. Autophagosomes are stable compared to other organelles in the cell, and autophagosomes only appear when needed [57]. Interestingly, during hibernation, the presence of well-developed autophagosomes within the cytoplasm of enterocytes and in the lumen of the intestine were observed (Figure 2B and Figure 8A,C). During long periods of starvation, too many autophagosomes developed. Eventually, the cytoplasm of the cell is filled with autophagosomes, and then the cell membrane ruptures, and organelles are discharged into the lumen of the intestine [11]. During the period of hibernation, more reserve bodies are gathered in the cytoplasm of enterocytes, which might be eventually removed through autophagy because this process performs a role in cell death [58]. On the other hand, the environmental stress of heat can deregulate the function of autophagy [59]. Taken together, our IHC, IF, and TEM data suggested that the activity of autophagy was decreased in the small intestine of *P. sinensis* during nonhibernation. Therefore, at low temperatures and during long periods of the hibernating state, the autophagic pathway is more active in maintaining cellular homeostasis by degrading cytoplasmic damaged organelles and proteins.

Previous reports have explained that mitophagy is the autophagic degradation of mitochondria, as a quality control mechanism for the maintenance of the internal cell environment [60]. This method is selective for degraded mitochondria that are isolated from fusion and fission processes [61]. In the current study, during hibernation, the enhanced level of PINK1 indicated more mitophagy. PINK1 is a mitophagy-related protein that is essential for cellular homeostasis under different circumstances [62]. Furthermore, during hibernation, the increased formation of multivesicular bodies was closely associated with autophagosomes. Recently, Fader reported that the process of degradation through autophagy required functional multivesicular bodies [63]. Multivesicular bodies are secreted by enterocytes for the maintenance of intestinal mucosal immunity [64]. Recently, studies have suggested that multivesicular bodies and autophagosomes may fuse together and create new vesicular structures, called amphisomes, then amphisomes fuse with the lysosome to digest the degraded material [11,65]. The autophagic lysosomal pathway inhibits the secretion of harmful proteins in the exosome for cellular fitness [66], and these autophagic vacuoles release vesicular luminal Ca^2+^ to facilitate interaction with multivesicular bodies [67]. As in our findings, the structures of the amphisome and exosome secretion pathways as well as the early endosome, late endosome, and multivesicular bodies were closely linked with autophagosomes inside the cytoplasm of enterocytes during hibernation (Figure 6C and Figure 8D). Therefore, reptilian intestinal enterocytes could be a good model to study the cross-talk between autophagosomes and multivesicular bodies.

## 5. Conclusions

**Figure 9:**
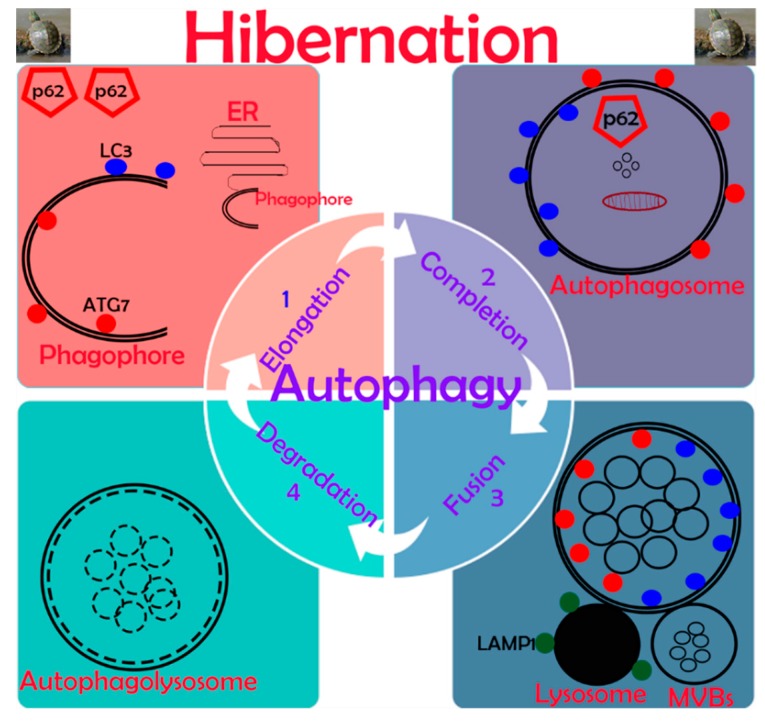
Schematic diagram of autophagy in enterocytes of the small intestine in *P. sinensis* during hibernation.

Our data demonstrate, for the first time, information about the in vivo up-regulation of autophagy in reptilian intestines during hibernation. Results indicated the expression of autophagy-specific markers provided solid evidence that autophagy is significantly active in the mucosal surface of the small intestine during hibernation. The cumulative formation of phagophores and autophagosomes enhance the autophagic pathway of micro-autophagy, macro-autophagy, and mitophagy in enterocytes of the small intestine for the maintenance of intestinal homeostasis of *P. sinensis*. A schematic diagram of autophagy in enterocytes of the small intestine of *P. sinensis* during hibernation is presented in Figure 9. We believe that our study opens a new avenue to unravel the significance of autophagy during hibernation.

## Figures and Tables

**Table 1:** Information about primary antibodies.

Primary Antibody	Species	Catalog No.	Dilution	Source
ATG7	Rabbit	10,088-2-AP	1:75	Proteintech
LC3	Rabbit	12,135-1-AP	1:75	Proteintech
P62	Rabbit	51,145	1:75	Cell signaling Tech
LAMP1	Rabbit	55,273-1-AP	1:75	Proteintech
PINK1	Rabbit	ab174775	1:1000	Abcam, USA
AMPK	Rabbit	Ab3759	1:1000	Abcam, USA

**Table 2:** Distribution of ATG7, LC3, p62, and LAMP1 in the mucosal region of the small intestine during hibernation and nonhibernation.

		ATG7		LC3		p62	LAMP1
		IHC	IF	IHC	IF	IF	IHC
**Hibernation**	**AC**	++	+++	+++	+++	+	++
	**BC**	++	+++	+++	+++	+	++
**Non-hibernation**	**AC**	+	+	++	+	+++	+
	**BC**	+	+	+	+	+++	-

(-) no expression; (+) weak, positive expression; (++) moderate, positive expression; (+++) strong, positive expression; IHC; immunohistochemistry, IF; immunofluorescence, AC; apical compartment, BC; basal compartment.

**Table 3:** The comparison of subcellular autophagy appearances in enterocytes of the small intestine of *P. sinensis* during hibernation and nonhibernation.

Enterocyte	Hibernation	Nonhibernation
Phagophore	++	+
Cytoplasmic Autophagosome	++	+
Luminal Autophagosome	+	-
Autophagolysosome	+	-
Mitophagy	++	-
Amphisome	+	-

More apparent (++), less apparent (+), and absent (-).
